# Trust dynamics in social media crises: a topic–aspect–sentiment analysis of public evaluations during a school food safety incident

**DOI:** 10.3389/fpsyg.2026.1895872

**Published:** 2026-08-05

**Authors:** Yaling Bai, Jiatong Tang, Baosheng Zhang

**Affiliations:** School of Economics and Management, Harbin Normal University, Harbin, China

**Keywords:** crisis communication, institutional trustworthiness, public evaluation, responsibility attribution, school food safety crisis, social media sentiment

## Abstract

School food safety crises often trigger intense discussion on social media and raise public concerns about institutional governance and public accountability. Yet existing studies have primarily focused on overall sentiment trends, providing limited insight into how public evaluations differ across specific governance issues or how sentiment is expressed toward these issues. Taking the 2024 spoiled meat incident at a middle school in Yunnan Province as a case, this study analyzes 11,016 Weibo posts and comments through a Topic–Aspect–Sentiment framework that combines LDA topic modeling, aspect extraction, and manual sentiment coding. The analysis examines how public evaluations were distributed across governance issues and how they evolved over the course of the crisis. The findings show that public evaluations were concentrated around governance issues related to food safety violations, response conduct, responsibility attribution, and accountability fairness rather than being evenly distributed across issues. Negative evaluations tended to be more concentrated during periods when response actions were perceived as inadequate or when responsibility was viewed as poorly assigned, with negative evaluations concentrated more heavily around these governance dimensions than around the food safety incident itself. During the remediation stage, relatively more favorable evaluations were observed in discussions surrounding regulatory interventions and corrective measures, although negative evaluations remained predominant overall during the observation period. In the later stages of the crisis, sentiment toward procedural legitimacy and accountability outcomes became relatively more balanced, reflecting continued public discussion concerning whether governance problems were being addressed through visible and credible procedures. By linking sentiment analysis to issue-specific evaluations, this study offers a more detailed account of how public evaluative patterns vary across governance issues in high-responsibility crises. The Topic–Aspect–Sentiment framework offers a practical approach for identifying evaluative signals in social media discussions and for examining how public evaluations shift across different issues and stages of a crisis. The findings also provide practical implications for information disclosure, accountability communication, response management, and long-term governance strategies in school food safety crises.

## 1 Introduction

In the digital media landscape, social media has become a key arena for public crisis communication. School food safety crises directly affect the health of minors and touch on the public's basic expectation that schools have a duty to protect students. Once such incidents come to light, public concern often goes beyond the food safety issue itself and is often interpreted as a failure of institutional management or routine oversight. The focus of public discourse then shifts from whether the food was unsafe to who should be held responsible and whether the institution can be trusted. This pattern of crisis communication—triggered by a food safety risk and amplified by blame attribution—distinguishes school food safety crises from ordinary food risk incidents. As such, these crises offer a valuable research context for examining the relationships among blame attribution, public sentiment, and perceived institutional trustworthiness.

Situational Crisis Communication Theory (SCCT) posits that the public's attribution of crisis responsibility directly affects how institutional actions are evaluated during a crisis. Among all crisis types, preventable crises that the public believes could have been avoided tend to be assigned greater responsibility (Coombs, 2007, 2022). School food safety crises fit this preventable crisis profile. Unlike externally triggered risks, school food safety problems typically arise within internal operational processes such as procurement, supply, and oversight. Consequently, the public is more likely to attribute the cause of such crises to the institution's failure to fulfill its responsibilities rather than to uncontrollable external factors. In this high-responsibility attribution context, the response strategies an institution adopts play a particularly significant role in shaping how the public judges its trustworthiness (Ma and Zhan, 2016). The Integrated Crisis Mapping model (ICM) further enriches this perspective from an emotional dimension and argues that public sentiment during a crisis is not simply a psychological reaction but a cognitive mechanism that shapes responsibility judgment and institutional evaluation (Jin et al., 2010). SCCT focuses on attribution logic, while ICM focuses on emotion structure. Together they form the theoretical basis for understanding how the public evaluates school food safety crises. However, existing studies tend to treat sentiment as an overall positive or negative tendency, and few examine within a single crisis event how different issues trigger distinct emotional structures. They also do not explain how issue-specific emotional responses become linked to public evaluations of institutional trustworthiness. In school food safety crises, the type and intensity of public sentiment vary across different issues such as regulatory accountability, information disclosure, and student health. Yet this kind of issue-level fine-grained analysis remains limited in existing research.

Building on this, the present study takes as its case the school food safety crisis triggered by the “spoiled meat incident” at a school in Yunnan province. Using 11,016 posts and comments collected from Weibo, the study constructs a Topic-Aspect-Sentiment analytical framework and systematically examines the core issues, sentiment distribution, and their effects on perceived institutional trustworthiness in school food safety crises. Rather than treating sentiment as an overall measure, this study understands it as a context-specific evaluative signal regarding particular issues. The clustering of sentiment across different issues reflects the public's dynamic judgments about institutional accountability, responsiveness, and governance reliability. From this perspective, the study treats public sentiment as an evaluative orientation directed at specific governance issues rather than as a direct indicator of institutional trust itself. Accordingly, the study focuses on the distribution of public evaluations of institutional response actions across different governance issues and examines their relationship with public perceptions of institutional trustworthiness. The implications for institutional trustworthiness are therefore discussed as broader interpretive implications derived from the findings rather than as directly measurable research outcomes.

Based on these issues, this study proposes three research questions.

RQ1: What are the core issues around which public discussion centers in school food safety crises?

RQ2: How does public sentiment vary in its distribution and in its stage-wise patterns across different issues?

RQ3: How do variations in sentiment across specific governance issues reveal public evaluations of institutional conduct during school food safety crises?

This study makes theoretical contributions in three areas. First, it situates school food safety crises within the preventable crisis category of SCCT and extends crisis communication research by showing how public evaluations of institutional conduct evolve across governance issues over the course of a crisis. Second, it develops a Topic–Aspect–Sentiment analytical framework that captures issue-specific sentiment distributions within a single crisis event, providing a fine-grained perspective that has been largely overlooked in existing crisis sentiment research. Third, by integrating topic modeling with manually validated aspect extraction and sentiment annotation, it provides an analytical approach for examining public evaluations expressed through social media discourse. At the practical level, this study also provides empirical insights for crisis communication and institutional governance in school food safety incidents.

## 2 Literature review

### 2.1 The preventable crisis nature of school food safety crises

School food safety crises are generally regarded as preventable, which means the public tends to assign responsibility to the institution from the outset. Educational crisis management research helps explain this attribution pressure. Because schools are expected to safeguard minors, crises in school settings are more likely than those in other organizations to trigger public questioning of institutional responsibility (Smith and Riley, 2012). Public concern extends beyond incident outcomes to sustained scrutiny of routine management practices. Consequently, evaluations of institutional trustworthiness in school food safety crises are closely tied to broader public judgments about whether institutions consistently fulfill their responsibilities. Under intense attribution pressure, the institution's response itself becomes a focal point of public evaluation. In preventable crises, responses that deflect responsibility or blur accountability are more likely to intensify negative public judgments, thereby making trustworthiness damage more difficult to repair (Coombs and Holladay, 2009). SCCT's attribution logic thus provides a theoretical basis for explaining why, in preventable crises, public judgments of institutional conduct tend to be expressed as assessments of institutional trustworthiness rather than evaluations of the incident itself. This dynamic has also been described as the politicization of crisis, whereby high-salience public crises often evolve from incident management into broader public contestation over accountability. How institutions define their role and communicate information directly shapes public judgments of governance legitimacy (Boin et al., 2017). In school settings, the moral sensitivity surrounding youth safety accelerates this process, allowing responsibility inquiries to spread more readily from specific incidents to sustained doubts about institutional trustworthiness as a whole. This is because public trust judgments are shaped not only by the objective food safety risk itself, but also by how food safety risks are perceived and interpreted within social media environments. Risk framing, information channels, and broader perceptions of institutional trustworthiness all play significant roles in shaping trust in regulatory institutions (Zhang et al., 2022). In the Chinese context, food safety issues are often deeply intertwined with the information environment, institutional accountability, and broader patterns of public trust (Han and Zhai, 2022). As a result, declining public trust is often cumulative and difficult to reverse through isolated or simplified responses. Existing research has largely examined school food safety through policy implementation or macro-governance perspectives, while paying comparatively less attention to how social media publics construct issue-specific responsibility judgments and how these judgments translate into trustworthiness evaluations of schools and regulatory authorities.

### 2.2 Sentiment analysis in crisis communication

Crisis communication research has long examined public responses during crises, with early studies often operationalizing these responses through sentiment orientation, typically categorized as positive, negative, or neutral. As social media has become a central platform for crisis information diffusion, sentiment analysis has been widely incorporated into crisis research because of its capacity to process large-scale, fragmented textual data. Particularly in high-salience public crises, fluctuations in sentiment polarity have been shown to effectively capture shifts in broader patterns of public evaluation, thereby serving as an important basis for crisis monitoring and governance decision-making (Che et al., 2022). However, crisis communication theory suggests that public reactions cannot be reduced to sentiment polarity alone. Within the Integrated Crisis Mapping (ICM) model, emotional expression in crises reflects more complex cognitive appraisal processes. It functions as a key mechanism through which the public assigns responsibility and evaluates institutions. Different crisis contexts activate distinct response patterns, which in turn shape judgments about the legitimacy of organizational responses (Jin et al., 2006, 2012). In social media environments, emotional expression extends beyond individual-level psychological reactions and becomes a communicative resource through which publics collectively evaluate institutional performance (Liu and Fraustino, 2014). This suggests that the value of sentiment analysis lies not merely in measuring sentiment distribution, but also in identifying the evaluative targets toward which sentiments are directed and the underlying judgment logics they reflect. At the conceptual level, distinguishing emotion from sentiment is equally important. Emotion typically refers to discrete psychological states such as anger, anxiety, or fear, whereas sentiment emphasizes the broader evaluative orientation embedded in text, making it more suitable for large-scale social media analysis (Acheampong et al., 2020; Mohammad, 2016). In this study, because the primary focus is on differences in evaluative orientation across issue-specific discussions on Weibo rather than on distinguishing discrete emotional categories, sentiment provides a more appropriate methodological framework than emotion. This study draws on the ICM model not to classify discrete emotional types, but to support the argument that public responses in crisis contexts carry evaluative significance—particularly as expressions of how the public judges institutional behavior. Although the study distinguishes only between positive and negative sentiment, these orientations still reflect the evaluative processes emphasized in the ICM framework, particularly those related to responsibility attribution and institutional assessment. Existing crisis communication research applying sentiment analysis has largely concentrated on aggregate polarity patterns, with comparatively limited attention to linking sentiment orientation to specific issue structures. However, aggregate sentiment measures tend to overlook the issue-specific structure of public evaluations in high-salience events. Even within a single crisis, negative sentiment surrounding different issues often reflects fundamentally distinct evaluative logics (Shaeri et al., 2025). Without integrating issue structure, sentiment analysis is limited in its ability to explain how perceptions of institutional trustworthiness are concretely formed.

### 2.3 Issue structure, sentiment distribution, and perceived institutional trustworthiness

Social media crisis research has increasingly recognized that public judgments of institutional trustworthiness are not formed through broad aggregate evaluations alone, but are constructed through sustained discussions around specific issues. In online public opinion crises triggered by extreme climate events, public trust has been shown to shift across crisis stages and to vary depending on the governance issues under discussion (Wang et al., 2025). The Social-Mediated Crisis Communication (SMCC) model emphasizes that crisis communication on social media has shifted from one-way information reception to multi-actor interaction. As publics seek information and engage in issue-based discussions, their understandings of responsibility are continuously reconstructed, making issue structure a central pathway through which crises are interpreted (Austin et al., 2012). Public interaction on social media can either strengthen or erode institutional trust, and this effect depends substantially on both the nature of the issues under discussion and the sentiment trajectories associated with them (Shah and Wei, 2022). Agenda-building and framing research further suggests that focal issues in crisis discourse significantly shape responsibility attribution and broader evaluative frameworks of governance (Entman, 1993). When crisis discourse centers on information concealment, delayed response, or responsibility avoidance, negative sentiment is more likely to evolve into systemic skepticism toward institutional transparency and governance capacity. Because perceptions of transparency independently and significantly shape public evaluations of governmental trustworthiness, information opacity itself becomes a direct source of institutional trustworthiness erosion (Grimmelikhuijsen and Meijer, 2014). Previous research has shown that perceived procedural transparency during crises predicts public trust, while the extent to which institutional responses address issues of greatest public concern shapes the effectiveness of governance communication (Coroado and Frateur, 2025). In school food safety crises, negative sentiment surrounding food sourcing may primarily reflect perceived risk, whereas negative sentiment surrounding regulatory negligence, school responses, or information disclosure is more likely to directly signal institutional responsibility and governance failure. What ultimately shapes the trustworthiness of schools and regulatory authorities is not merely the presence of negative sentiment, but the issue domains in which such sentiment becomes concentrated. This study treats issue-level sentiment as a form of public evaluation directed at institutional behavior: when sentiment concentrates around issues such as responsibility attribution or information transparency, it reflects how the public appraises different dimensions of governance. Sentiment distribution can therefore reveal patterns of public evaluation rather than trust itself. However, existing research continues to focus primarily on aggregate opinion fluctuations or crisis response strategies, offering limited fine-grained analysis of how issue structure, sentiment distribution, and perceived institutional trustworthiness interact within a single crisis event. Accordingly, this study conceptualizes governance issues as an interpretive layer through which differences in public sentiment can be linked to evaluations of institutional trustworthiness. Using a Topic–Aspect–Sentiment framework, the study examines cross-issue variations in sentiment distribution to reveal how evaluations of institutional trustworthiness emerge during school food safety crises.

### 2.4 Applications and limitations of computational methods in crisis communication research

As social media data has expanded dramatically, computational methods have become central tools in crisis communication research. Topic modeling, sentiment analysis, and machine learning are now widely used to identify discourse structures and shifts in public attitudes during crises (Reuter and Kaufhold, 2018). On short-text platforms such as Twitter and Weibo, LDA-based topic modeling and sentiment classification have been extensively used to identify crisis-related issue structures and sentiment trajectories, enabling researchers to trace both issue evolution and shifts in public evaluative orientation across crisis stages. Recent studies further demonstrate the effectiveness of computational approaches in crisis framing analysis and in revealing evolving patterns of issue attention and public sentiment throughout crisis development (Figueiredo, 2024; Nguyen et al., 2024). However, the principal strength of existing computational methods lies in identifying salient discussion topics and aggregate sentiment direction. Their explanatory capacity remains limited when addressing why specific evaluations emerge around particular issues and how those evaluations develop into judgments of institutional responsibility and trustworthiness. In complex public controversies, sentiment classification often succeeds in detecting polarity while failing to specify evaluative targets. A text classified as negative does not necessarily reveal whether criticism is directed at the event itself, responsible actors, or governance procedures (Mohammad, 2021). Although existing studies increasingly explore relationships between crisis response strategies and public sentiment expression, they have paid comparatively less attention to the internal issue–sentiment architecture within single crisis events (Zhong et al., 2024). Systematic reviews of social media crisis communication research similarly indicate that much of the field relies on broad sentiment categories or cross-event comparisons, with insufficient fine-grained analysis of internal dynamics within individual crises—an especially significant limitation in contexts where institutional accountability is highly salient (Cheng et al., 2022). At the same time, automated text analysis that lacks theoretical grounding and manual validation risks producing methodological sophistication without corresponding interpretive depth (Jaidka et al., 2020). In institutionally sensitive crises such as school food safety incidents, reliance on automated coding alone is particularly insufficient for capturing how publics construct trustworthiness judgments toward responsible institutions. To address this limitation, this study integrates computational identification with manual validation. LDA is employed to identify core topic structures, which are then refined through manual sentiment coding and aspect classification. This design preserves sensitivity to contextualized expression, irony, and crisis-specific discourse while reducing the risk that automated classification oversimplifies complex governance evaluations. The methodological contribution of this study does not lie in advancing sentiment detection technology itself, but in developing a Topic–Aspect–Sentiment framework that integrates the scalability of computational methods with the contextual interpretive strength of manual coding. In doing so, it more precisely captures how publics form evaluations of institutional responsibility and trustworthiness around specific issues, thereby addressing a central methodological gap in crisis communication research between automated scalability and governance-oriented explanatory depth.

### 2.5 Summary of literature and research gaps

Despite substantial advances in understanding the institutional accountability dimensions of school food safety crises, public sentiment expression on social media, and the role of such expression in shaping institutional trustworthiness judgments, gaps remain in explaining how public sentiment around school food safety crises contributes to evaluations of institutional trustworthiness. First, although prior research has established the theoretical framing of school food safety crises as preventable crises, little systematic attention has been paid to how publics develop differentiated patterns of sentiment concentration around distinct governance issues in social media contexts. Existing discussions of attribution pressure have largely remained at the macro level, with limited examination of issue-specific sentiment structures within individual crisis events. Second, although crisis communication scholarship recognizes the evaluative function of sentiment expression, methodological applications remain largely confined to aggregate polarity statistics, with insufficient integration of sentiment orientation and issue structure. Third, computational communication research broadly faces the limitation of unclear evaluative targets, as automated sentiment classification often struggles to capture context-specific public judgments of institutional trustworthiness. By contrast, manual coding offers greater sensitivity to context and crisis-specific discourse, thereby helping address this methodological gap and improving interpretive validity in fine-grained analyses of issue–sentiment structures. These three gaps converge on one central question: how does sentiment distribution across specific governance issues relate to public evaluations of institutional trustworthiness in school food safety crises, and how can this relationship be systematically examined within a single crisis? To address this question, the present study develops a Topic–Aspect–Sentiment framework that uses LDA to identify core topic structures while incorporating manual sentiment coding to preserve contextual interpretive depth. This design enables a systematic examination of how issue structure, sentiment distribution, and perceived institutional trustworthiness interact within a single crisis event.

## 3 Research design

### 3.1 Case selection and event overview

#### 3.1.1 Rationale for case selection

This study examines the 2024 “spoiled meat incident” at a middle school in Yunnan Province because it offers three analytically significant features: a clearly traceable institutional accountability structure, sustained multi-issue discussion on social media, and a relatively complete crisis response cycle. Together, these characteristics make the case well suited for examining key stages in the formation of public evaluations of institutional trustworthiness during school food safety crises.

Within China's school meal system, food provision extends beyond individual schools and operates through a multi-level governance structure involving schools, contractors, education authorities, and market supervision agencies. Local education bureaus and public health authorities bear formal responsibilities for oversight, inspection, and corrective action. This governance arrangement means that food safety failures are not confined to schools themselves, but readily extend to evaluations of whether local regulatory bodies have fulfilled their institutional responsibilities, thereby directly linking governance failures to public judgments of institutional trustworthiness.

On Weibo, the incident generated sustained discussion across multiple governance-related dimensions, including food quality, school management, official response, investigative transparency, and accountability measures, thereby providing a rich context for examining how different issues correspond to distinct sentiment orientations. Moreover, the incident unfolded over a relatively compressed timeframe that included exposure, official response, investigation disclosure, accountability enforcement, and remediation. This clearly delineated governance sequence allows for stage-by-stage alignment between online discourse and crisis management processes, while reducing analytical distortion from later-stage narrative reversals. The single-case design was chosen to examine the relationships among issue structure, sentiment distribution, and public evaluation within an empirically rich context. As a high-responsibility crisis involving schools, regulatory authorities, and catering contractors, the incident exhibits governance characteristics that are broadly representative of school food safety crises. While the findings cannot be generalized directly to all crisis contexts, the analytical framework developed here has broader relevance for institutionally sensitive crises in which public evaluation is particularly salient.

#### 3.1.2 Event overview

On October 16, 2024, a parent at a middle school in Kunming, Yunnan Province discovered what appeared to be spoiled meat in the school cafeteria and alerted police. Images and videos from the scene quickly circulated across social media, thrusting concerns over school food safety into public scrutiny. Initial public discussion focused primarily on food quality and potential health risks to students.

On October 17, the district Bureau of Education and Sports, together with the Market Supervision Bureau, issued an official statement launching an investigation, while the school arranged for the cook involved to deliver a public apology. However, during a meeting with parents later that day, school and education officials left before parents had fully expressed their concerns. Footage of the incident circulated widely across media and social platforms, redirecting public scrutiny from spoiled food itself toward institutional response, administrative attitude, and accountability.

Between October 19 and 20, the district joint investigation team released its findings and imposed disciplinary measures on the catering contractor and responsible individuals involved. Schools across the district simultaneously initiated targeted inspections and corrective actions. Subsequently, the municipal Party Standing Committee further strengthened school food safety oversight. Online discourse increasingly centered on the adequacy of penalties, the scope of accountability, and the trustworthiness of long-term corrective mechanisms.

**Figure 1:**
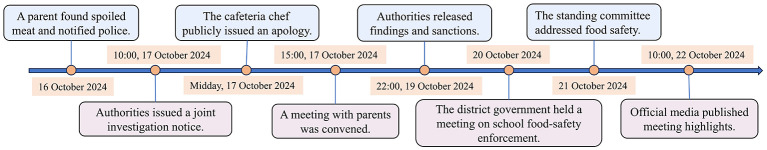
Timeline of the public opinion event.

Overall, the incident evolved from an isolated food safety event into a broader grassroots governance crisis centered on emergency response, accountability, and regulatory failure. Each stage was associated with distinct issue priorities and patterns of responsibility attribution, creating an analytically structured empirical setting for examining how issue structure and sentiment distribution jointly shape perceptions of institutional trustworthiness. Figure 1 presents the event timeline.

### 3.2 Analytical framework

This study develops a Topic–Aspect–Sentiment analytical framework and draws on relevant Weibo posts and comments as its textual corpus. This framework is based on the premise that public sentiment in school food safety crises does not manifest uniformly across the crisis as a whole, but instead clusters into differentiated evaluative patterns around distinct governance issues. In such crises, analyzing sentiment distribution across specific issues offers greater explanatory power for understanding public responses to different governance dimensions than aggregate sentiment polarity statistics alone. Importantly, this study does not equate online sentiment with institutional trustworthiness itself, nor does it treat sentiment outcomes as a direct measure of trustworthiness. Rather, this study examines how sentiment distribution across different issues functions as an evaluative signal through which publics express perceptions of institutional response performance, accountability implementation, and governance execution. Accordingly, online sentiment is treated not as a proxy for institutional trustworthiness itself, but as an analytical lens through which public evaluations of institutional trustworthiness can be examined.

**Figure 2:**
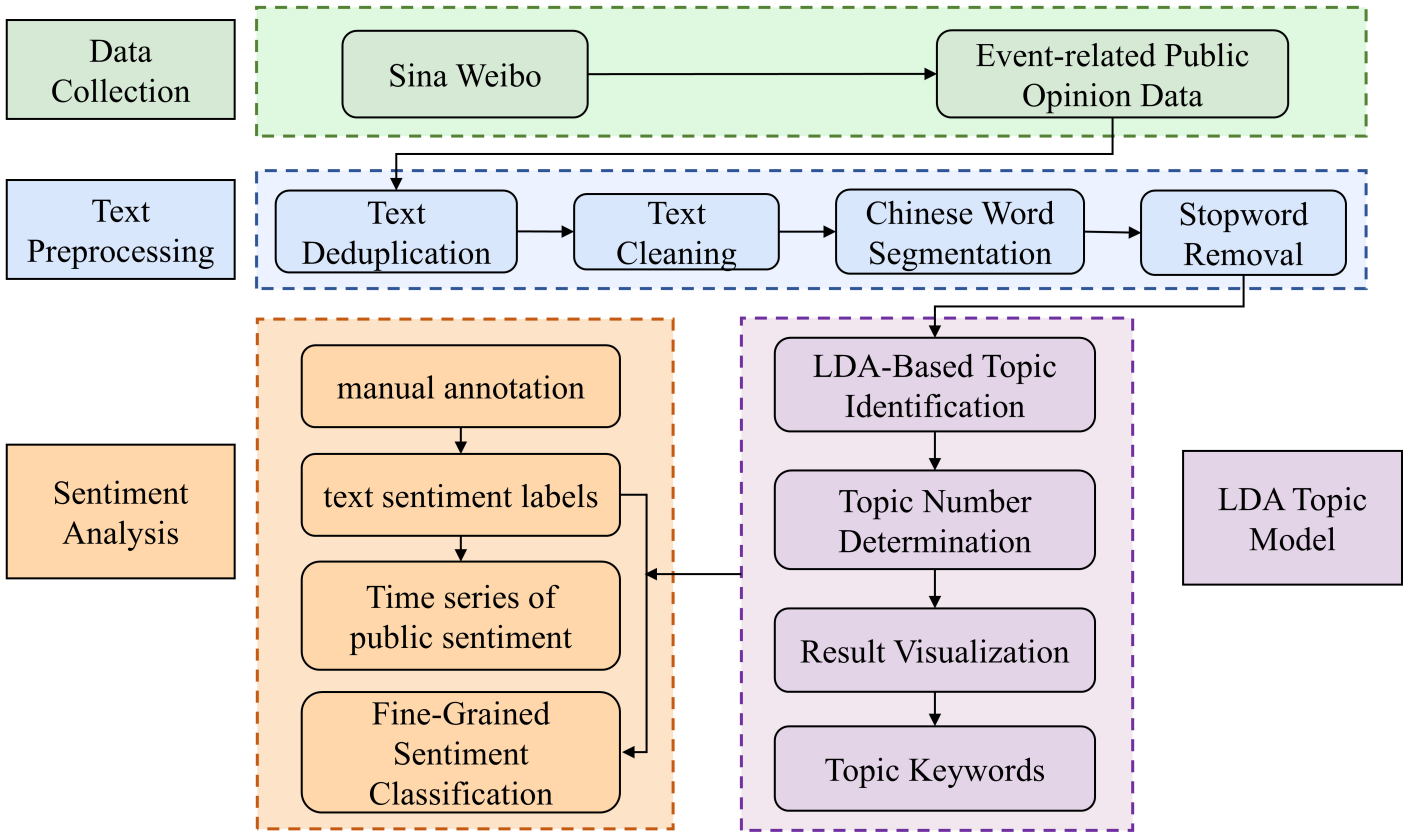
Overall analytical procedure.

The study's analytical design is conducted at three analytical levels. First, all texts are manually coded for sentiment to trace the overall trajectory of public sentiment across the exposure, escalation, response, and follow-up stages of the crisis, thereby establishing a stage-specific contextual foundation for subsequent analysis. Second, LDA topic modeling is employed to identify the dominant topic structure of event-related discussion, organizing dispersed online discourse into coherent issue clusters in order to identify the primary focus of public attention. Third, building on topic identification, specific governance aspects within each topic are refined through contextual semantic analysis, with particular attention to responsibility attribution, response processes, information disclosure, and institutional arrangements. These aspect-level dimensions are then integrated with manually coded sentiment data to examine differentiated sentiment patterns within specific issues. By integrating these three levels, the analysis bridges macro-scale sentiment trajectories and topic structures with micro-scale evaluative dynamics, offering a granular look at how emotions shift within specific issues. This framework maps public sentiment across various governance nodes and tracks its temporal evolution. This approach reveals how evaluations of institutional trustworthiness vary across governance issues and different stages of a crisis. Incorporating the aspect level is analytically important because a single topic often contains multiple distinct evaluative targets. Texts assigned to the same topic may address institutional actors, response measures, and information disclosure at the same time. Analyzing sentiment at the topic level alone risks obscuring meaningful differences in how the public evaluates these distinct objects. The aspect level distinguishes among these evaluative targets, providing a more precise basis for examining how sentiment is distributed across specific governance issues. For conceptual clarity, this study distinguishes among three terms used at different analytical levels: topic, aspect, and issue. Topic refers to the thematic clusters identified through LDA topic modeling and captures the overall structure of public discourse surrounding the event. Aspect refers to the fine-grained evaluative dimensions identified within each topic and functions as the core analytical unit for examining variations in public sentiment. Each aspect represents a specific actor, response behavior, or governance process toward which public evaluations are oriented. Issue corresponds conceptually to aspect but is employed primarily as an interpretive construct, emphasizing the broader governance significance of these evaluative dimensions within public discourse. In short, topic identifies what the public is discussing, whereas aspect and issue explain how those discussions are specifically evaluated. Throughout the analysis, attention is focused on patterns of public evaluation reflected in sentiment distribution across governance issues and on how these patterns evolve over the course of the crisis. References to institutional trustworthiness in the analysis and discussion are used to identify the targets toward which public evaluations are directed; they do not constitute direct measurements of public trust or institutional trustworthiness. Figure 2 illustrates the overall analytical procedure.

## 4 Empirical results

### 4.1 Data collection and processing

This study uses public discussions on Sina Weibo related to the spoiled meat incident at a middle school in Yunnan Province as its primary data source. Data were collected through web scraping using keyword combinations directly related to the incident, including “Yunnan middle school spoiled meat,” “Kunming school cafeteria,” and “Kunming school food safety,” to ensure that the corpus focused on this event rather than on school food safety discussions more broadly. Different keyword combinations may produce variations in corpus composition, which may in turn influence topic modeling results. To reduce this limitation, the study employed a combined search strategy using multiple complementary keywords and drew from institutional accounts, media accounts, and individual users to broaden the range of sources represented in the corpus. The dataset included posts and comment threads from institutional accounts central to the event (e.g., Kunming Guandu Release), related discussions initiated by major media accounts (e.g., CCTV News and People's Daily), and posts and comments from highly visible individual users. Data were collected between October 16 and October 28, 2024, resulting in 19,817 raw texts.

The dataset included two forms of content: posts published by institutional or media accounts and comments produced by ordinary users. While the former primarily conveyed official information and public positioning, the latter served more directly as a space for public sentiment and opinion expression. Because this study focuses on the broader issue structure and sentiment distribution of public discourse rather than source-specific communication forms, both were analyzed together within a unified corpus. Pure reposts without added original content were excluded during preprocessing.

After removing duplicates, irrelevant material, and invalid text elements, 11,016 valid texts were retained for analysis. Chinese text segmentation was conducted using jieba, followed by stopword filtering. The stopword list combined the Harbin Institute of Technology and Baidu stopword dictionaries, with additional event-specific refinements in two areas. One consisted of Weibo-specific non-semantic markers such as “expand full text,” “image comment,” and “Weibo video.” The other included high-frequency but low-information event-related terms, such as geographic identifiers (Yunnan, Kunming, Yunnan Province), the event label itself, and common filler expressions. These refinements improved topic clarity and helped the LDA model better capture substantively meaningful themes. The final stopword list contained 1,720 entries.

### 4.2 Ethical considerations and data privacy

Data for this study came exclusively from publicly accessible Sina Weibo posts and comments related to the Yunnan school spoiled meat incident. No private messages or restricted-access content were included. During preprocessing, usernames, profile links, and other directly identifying information were removed, and only anonymized textual content and timestamps were retained for analysis. No direct contact with users occurred, and the research involved no intervention in online discussions. Consistent with common ethical guidance for social media research, secondary analysis of anonymized public posts is often considered exempt from formal institutional review requirements. To further minimize re-identification risk, only aggregated findings are reported, and no rare, traceable, or highly context-specific posts are reproduced.

### 4.3 Overall sentiment trajectory

All 11,016 texts were manually coded for sentiment. A binary positive–negative sentiment scheme was adopted for two reasons. First, the study compares the direction of public evaluation across different governance issues, and classifying texts according to their dominant evaluative orientation provides a consistent basis for comparison. Second, this approach is consistent with prior social media research on school food safety and public crises (Liu et al., 2023), thereby enhancing comparability across studies. Negative sentiment was assigned to texts expressing dissatisfaction, criticism, skepticism, or concern, while positive sentiment captured expressions of approval, support, or favorable evaluation. Prior to formal coding, two coders jointly revised the coding manual using a randomly selected sample of 1,000 texts and discussed borderline cases until a shared understanding of the coding criteria was reached. The two coders then independently coded the full dataset without access to each other's annotations. For borderline cases combining factual description with evaluative content, or expressing conditional or ambiguous opinions, coders classified the texts according to their dominant evaluative orientation. Cases that remained unresolved after discussion were referred to a third coder for a final decision. Inter-coder reliability was assessed using Cohen's κ (κ > 0.75), indicating substantial agreement under the Landis and Koch standard (Landis and Koch, 1977).

**Figure 3:**
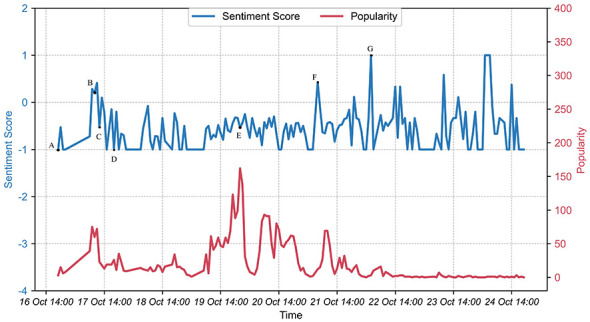
Comparison between sentiment scores and popularity metrics. The labels **A–G** indicate the chronological sequence of key events in the crisis timeline: **A**, Parent reported to the police (16 Oct); **B**, Investigation initiated (17 Oct 10:00); **C**, Chef issued an apology (17 Oct Midday); **D**, Press meeting interrupted early (17 Oct 15:00); **E**, Official disciplinary decision released (19 Oct 22:00); **F**, Special rectification work arranged (21 Oct); **G**, Municipal Standing Committee ordered follow-up measures (22 Oct).

Stage classification was informed by Fink (1986) crisis life cycle framework—prodromal, acute, chronic, and resolution—while also incorporating shifts in Weibo discussion intensity and identifiable official response milestones. Stage 1 (exposure) began with parental discovery of the incident and its rapid spread on social media. Stage 2 (acute escalation) was marked by the public apology and officials' early departure from the parent meeting. Stage 3 (investigation and remediation) centered on the release of district-level findings and subsequent corrective meetings. Stage 4 (post-event decline) reflected the gradual cooling of online attention following further directives from municipal authorities. Figure 3 illustrates the overall trajectory of sentiment scores and event-related attention. This four-stage structure was designed to capture stage-specific shifts in public sentiment as governance responses unfolded, particularly the temporary moderation observed during Stage 3. Such variation can be obscured in simpler two- or three-stage models, despite being important for understanding how public sentiment changed alongside major governance responses.

Stage 1 (October 16 to the morning of October 17) marked the exposure and initial diffusion phase of the crisis. Following a parent's on-site discovery, the incident spread across social media, generating early negative sentiment, though overall volume remained limited. During this period, authorities released preliminary investigative information promptly; some users appeared to adopt a wait-and-see stance, and overall sentiment remained comparatively stable.

Stage 2 (the afternoon of October 17 to the afternoon of October 20) exhibited the most pronounced shift in sentiment across the crisis period. The cook's public apology alone, together with the early departure of school and departmental representatives from the parent meeting, coincided with a period of rapid escalation in online discussion. Public attention appeared to shift from food safety itself to the adequacy of institutional responses and responsibility attribution during this period. Sentiment scores declined sharply, discussion volume peaked, and negative sentiment became dominant.

Stage 3 (the afternoon of October 21 to the morning of October 22) showed a brief moderation in sentiment. During the period when district-level investigation findings were released and remediation meetings were held, positive sentiment rose modestly. Sentiment scores partially recovered, while online discussion volume began to contract.

Stage 4 (the afternoon of October 22 to October 24) was characterized by a continued decline in overall discussion volume. However, intermittent disputes over penalty severity and accountability scope persisted. Residual negative sentiment remained, and the overall distribution had not yet returned to neutrality.

Overall, spikes and diffusion in negative sentiment were tightly coupled with critical response junctures, notably the quality of official response conduct and the progression of accountability processes. Officials' early departure coincided with a sharp decline in sentiment scores, whereas periods of targeted remediation were associated with a temporary rebound. Taken together, these patterns indicate that changes in sentiment distribution coincided with shifts in public discussion about institutional responsiveness and responsibility. Prior research suggests that the public often weighs negative cues more heavily than positive ones, which may help explain why, in this case, institutional responses appeared to attract more critical attention than the triggering incident itself (Robertson et al., 2023; Schöne et al., 2021; Soroka et al., 2019).

However, overall sentiment trajectories capture only broad patterns of public opinion and cannot distinguish how sentiment varies across specific governance issues. To clarify where negative sentiment was most concentrated and how public evaluations varied across governance issues, the subsequent analysis shifts to fine-grained topic–aspect sentiment patterns.

### 4.4 Topic–aspect–sentiment analysis

#### 4.4.1 Topic structure

**Figure 4:**
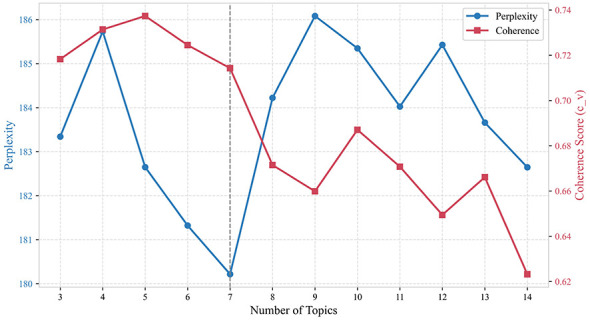
Perplexity curve of the LDA model.

This study uses Latent Dirichlet Allocation (LDA) to identify topic structure within the Weibo corpus. LDA was selected for two reasons. First, the corpus-level modeling strategy, rather than single-post analysis, helps mitigate the sparsity commonly associated with short social media texts. Second, prior research on Chinese social media discourse has shown LDA to offer practical interpretability for identifying coherent issue structures. To improve text length and contextual density, comments posted by the same user within proximate time windows were merged prior to modeling. Model implementation followed established computational communication practices in public relations research (Britt et al., 2025; Zhou et al., 2023), using multiple evaluative criteria across topic selection, stopword construction, and manual validation stages. Candidate models across K ∈ [3,14] were iteratively tested, with perplexity and topic coherence used jointly to assess statistical fit and semantic interpretability (see Figure 4). Perplexity reached its minimum at K=7, while coherence remained comparatively high before declining beyond this point, suggesting that K=7 provided the most analytically stable balance between fit and interpretability. To complement these quantitative metrics, we manually scrutinized high-frequency keywords across candidate values of K, evaluating their inter-topic distinctiveness and intra-topic coherence to validate K=7 as the optimal solution.

**Figure 5:**
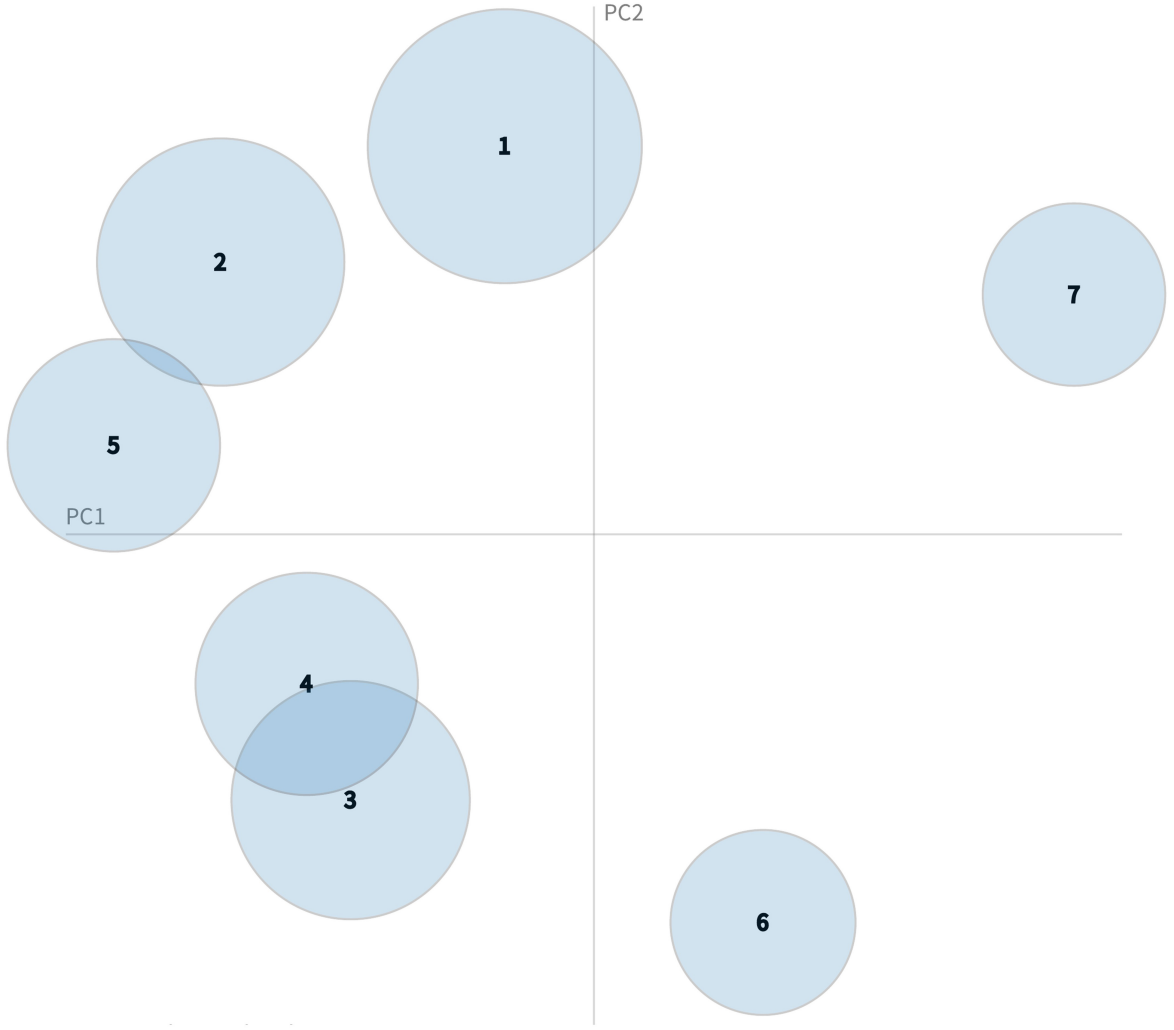
Intertopic distance map.

To assess semantic distinctiveness across topics, pyLDAvis was used to generate an intertopic distance map (see Figure 5). The seven topics appeared relatively dispersed in two-dimensional space, indicating satisfactory overall differentiation in semantic structure. Although Topic 3 and Topic 4 showed partial overlap in the intertopic distance map, manual inspection of their high-frequency keywords revealed substantive differences in semantic focus. Topic 3 was anchored by terms such as “law,” “fine,” “illegal,” “penalty,” and “bottom line,” reflecting public discussion of the severity of legal sanctions and the boundaries of accountability. Topic 4 was defined by terms such as “attitude,” “arrogance,” “threat,” “tuition fees,” and “exposure,” pointing to criticism of school governance conduct and the interests underlying school–contractor relations. The former centered on whether punishment outcomes met legal expectations; the latter focused on whether institutional behavior and power arrangements were appropriate. Merging the two would conflate these distinct evaluative logics, obscuring differences in how the public assessed different governance dimensions and reducing the explanatory value of fine-grained analysis.

Following iterative review and validation, the seven topics were interpreted as: official notification, responsible actors, legal and disciplinary responses, school governance, food safety concerns, conflicts of interest, and broader societal remediation (see Table 1).

**Table 1:** Topics and topic feature words.

Topic	Topic feature words
Topic 1	Official, notification, spoiled meat, investigation, case registration, unpleasant smell, situation report, market regulation, administrative bureau, investigation of meal provision.
Topic 2	Principal, company, education bureau, leaving the meeting, vice principal, enterprise, meeting with parents, government agency, government, contractor.
Topic 3	Society, state, law, people, bottom line, fine, illegal, penalty, capitalists, cost.
Topic 4	Education, attitude, school authorities, arrogance, threat, news, tuition fees, exposure, enterprise, corporation.
Topic 5	Cafeteria, supervision, spoilage, market, testing, transportation, operations, spoiled meat, private school, processing.
Topic 6	Chef, leadership, interests, officials, capital, ordinary citizens, person in charge, take the blame, supplier, question.
Topic 7	Regulation, responsibility, implementation, institution, meal accompanying system, catering, intensity, diet, rectification, mechanism.

#### 4.4.2 Aspect extraction and text segmentation

Aspect extraction builds on the LDA topic structure by providing a more fine-grained level of analytical differentiation. LDA-derived topics capture thematic clusters at the corpus level, yet each topic often contains evaluations directed toward multiple actors, behaviors, or governance processes. Sentiment analysis conducted solely at the topic level therefore risks masking important intra-topic variation across distinct evaluative aspects. For example, within Topic 2, public evaluations targeted multiple distinct objects, including school leadership, education authorities, response measures, and school–contractor relations, rather than a single evaluative target. This aspect-level distinction is important because the analysis focuses on how public evaluations differ across governance issues rather than on inferring broader changes in institutional trust. Collapsing these into a single analytical unit would obscure meaningful differences in public evaluation. Accordingly, specific aspects were extracted within each topic to align sentiment analysis more closely with concrete governance issues.

Aspect extraction began with high-frequency keywords within each topic and incorporated contextual semantic reference to determine evaluative focus. Aspect extraction followed a three-step procedure. In the first step, the top 30 high-frequency keywords identified by LDA for each topic were compiled as candidate terms. In the second step, two researchers independently reviewed the candidate terms and identified those referring to governance actors or processes—such as schools, regulatory authorities, supply chains, accountability measures, and investigation procedures—as candidate aspects. Semantically similar terms referring to the same evaluative target were then merged, whereas ambiguous terms or those only weakly related to governance themes were removed. In the third step, the two researchers cross-checked their independently generated aspect lists and resolved disagreements through discussion until consensus was reached. Because aspects were inductively constructed at the category level rather than assigned to individual texts, the credibility of the extraction process was established through independent coding, cross-checking, and consensus building rather than formal intercoder reliability statistics. This process yielded 28 analytically distinct aspects with clear referential targets across the seven-topic framework. Texts were then segmented into coded semantic units, each linked to a specific topic and aspect, thereby connecting the topic–aspect structure to manually coded sentiment data and establishing the basis for subsequent fine-grained analysis (see Table 2).

**Table 2:** Topics and core entities.

Topic	Core Entities
Topic 1	Official investigation, notification/official notification, food safety, market regulation.
Topic 2	Principal/vice principal, enterprise/company, education bureau/government, response measures.
Topic 3	Society/state, capital conflict, legal baseline, punishment/fine.
Topic 4	Response attitude, fee disputes, school–enterprise tensions, issue exposure.
Topic 5	Spoiled meat, supply chain, operational management, quality control.
Topic 6	Leadership/officials, capital/interests, key actors, public reaction.
Topic 7	Regulatory mechanism, social rectification, canteen/dining, responsibility implementation.

#### 4.4.3 Topic–aspect–sentiment classification results

**Figure 6:**
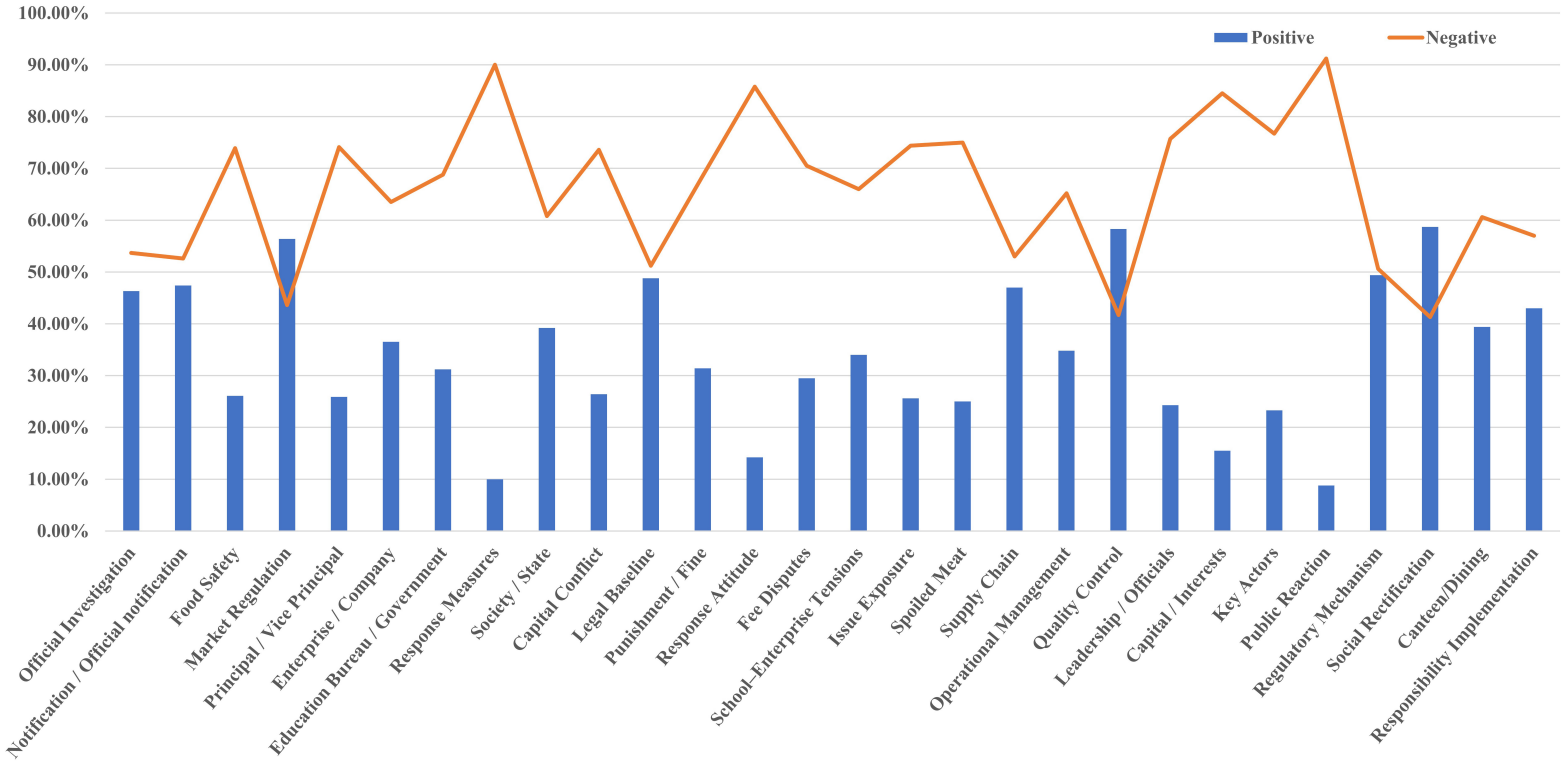
Proportion of sentiments across topics.

Drawing on manually coded sentiment data, this study analyzed sentiment polarity across 28 aspects within the seven-topic framework, calculating the proportion of positive and negative sentiment for each aspect to examine variation in public evaluation across governance dimensions (see Figure 6). To assess the extent to which sentiment distribution within each aspect deviated from a neutral proportional baseline, Cohen's h was calculated for each aspect using 0.5 as the reference point (Cohen, 1988). Compared with simple proportion comparisons, this approach provides a more stable estimate of sentiment deviation magnitude. The term “aspects with a relatively balanced sentiment distribution,” as used in subsequent analysis, does not refer to a separate sentiment coding category. It is instead an aggregate-level statistical description based on effect size. When Cohen's h for an aspect is close to zero (|h| < 0.2), the proportional difference between positive and negative texts is small, indicating that neither sentiment orientation is clearly dominant. This description reflects the distributional balance between the two categories of coded texts within a specific aspect rather than a direct characterization of any individual text's sentiment.

The results indicate substantial differentiation in sentiment distribution across the 28 aspects. Nine aspects exhibited large effect sizes (|h| > 0.5), with sentiment strongly skewed negative. These were concentrated primarily in accountability-related domains, including response attitudes, public reactions, and commercial conflicts of interest. Ten aspects fell within the moderate range (0.2 < |h| ≤ 0.5), displaying a clear but less intense negative skew. Six aspects showed effect sizes near zero (|h| < 0.2), reflecting relatively balanced distributions of positive and negative sentiment. These included official investigation, official notification, legal baseline, supply chains, responsibility implementation, and regulatory mechanisms. Such aspects are referred to in this study as having a relatively balanced sentiment distribution. Three aspects—market regulation, quality control, and social remediation—produced negative h values, indicating a relative shift toward positive sentiment. This pattern indicates that aspects associated with regulatory intervention and institutional correction tended to receive relatively more favorable evaluations.

At the aggregate level, issues directly tied to governance capacity, accountability, and official conduct were more likely to attract strong negative evaluations, whereas institutional arrangements and subsequent remediation measures tended to receive comparatively moderate assessments. This pattern suggests that public sentiment was not uniformly distributed across the crisis but instead clustered around different governance issues. These differences provide the basis for the subsequent analysis of how public evaluations varied across governance issues. The sentiment distribution patterns identified in this study reflect how the public evaluated specific governance issues during the crisis; references to institutional trustworthiness in the discussion serve an interpretive purpose only.

## 5 Discussion: fine-grained sentiment structure and public evaluations of governance issues

### 5.1 Main findings and implications for crisis communication

This study demonstrates that shifts in public sentiment during school food safety crises are shaped not solely by the triggering food safety event, but by the evolving interaction of responsibility attribution, response conduct, and subsequent institutional handling. Overall, public sentiment followed a discernible stage-based trajectory. During the initial exposure stage, discussion focused primarily on food safety risk itself, with sentiment largely negative yet comparatively restrained. As response failures and responsibility disputes emerged, public attention rapidly shifted from the existence of the problem to the way it was managed, producing a marked intensification of negative sentiment. Following investigation, remediation, and regulatory intervention, negative sentiment partially moderated. However, negative evaluations that emerged during earlier stages continued to appear in later discussions, particularly regarding accountability and governance performance. In the final stage, low-intensity negative evaluations surrounding accountability, fairness, and institutional enforcement remained present.

Beyond differences across crisis stages, sentiment was also unevenly distributed across governance issues, gradually consolidating around relatively stable evaluative patterns focused on food safety failures, response posture, responsibility implementation, and institutional enforcement. The findings suggest that inadequate responses, imbalanced responsibility allocation, and insufficient penalties were consistently associated with higher concentrations of negative sentiment. As the crisis evolved, this negativity extended beyond criticism of the incident itself to broader evaluations of institutional conduct and governance performance. By contrast, regulatory intervention and remediation coincided with some reduction in public pressure. However, this effect appeared limited: negative evaluations established during the earlier stages were not substantially altered by the more favorable evaluations that emerged during remediation. Taken together, these patterns indicate that in school food safety crises, public sentiment extends beyond immediate reactions to the event itself and instead reflects continuing judgments about governance processes, institutional performance, and accountability arrangements.

Theoretically, these findings enrich established crisis communication frameworks. SCCT conceptualizes preventable crises as high-attribution situations. This study further suggests that under social media conditions, such attribution pressure is expressed not merely through rising overall negativity, but through concentrated sentiment around particular governance issues—especially response posture and responsibility attribution. While the ICM model emphasizes that public responses evolve dynamically throughout crises, the present findings suggest that changes in public evaluation are closely associated with shifts in issue salience across different crisis stages. This study shows that such shifts occur not only across crisis types, but also within a single crisis as issue salience changes over time. From the perspective of crisis politicization, school food safety crises are more than service failures; under conditions of intense public attention, they can evolve into public accountability processes centered on who should be held responsible. In this context, behaviors such as officials leaving early or penalties perceived as insufficient become focal triggers of concentrated negative sentiment.

Importantly, this study does not equate online sentiment with institutional trustworthiness, nor does it claim to measure trustworthiness directly. Instead, sentiment distribution across various issues is treated as an evaluative signal reflecting public judgment on governance. The analysis shows how public evaluations of institutional performance varied across governance issues throughout the crisis. The following sections therefore examine three related dimensions: how negative sentiment becomes concentrated around specific governance issues, how positive evaluations emerge under particular governance conditions, and how relatively balanced sentiment distributions reflect continuing public evaluation of institutional processes.

### 5.2 Negative sentiment: food safety failures, response missteps, and insufficient accountability

Fine-grained analysis indicates that during the acute crisis stage, negative sentiment was not evenly distributed across issues but concentrated around four dimensions: the nature of the food safety violation itself, response handling, the perceived fairness of interest and governance structures, and the adequacy of subsequent accountability. More consequential than the immediate reaction to the food safety problem itself was the way public attention gradually shifted toward broader criticism of institutional response capacity and governance performance. This pattern aligns with SCCT's core argument that preventable crises generate high responsibility attribution: when a crisis is perceived as avoidable, public judgment more readily expands from the incident itself to broader evaluations of institutional accountability and governance capacity.

Food safety concerns constituted the most immediate source of negative sentiment. Aspects related to food safety, spoiled meat, and school catering consistently attracted high levels of negative sentiment, underscoring the exceptional sensitivity of food safety failures in school settings involving minors. Such problems were rarely interpreted as isolated operational failures; instead, they were more often framed as evidence of regulatory breakdown or a fundamental breach of institutional responsibility. Here, the fact that the issue was first identified and circulated by parents rather than detected through internal management or formal oversight mechanisms substantially reinforced perceptions of routine supervisory failure. High-profile precedents such as the “Rat-head, Duck-neck” scandal further strengthened this association through anchoring effects, making the public more likely to interpret new incidents through preexisting frameworks of institutional negligence (Tversky and Kahneman, 1974).

Response failures were associated with particularly high concentrations of negative sentiment surrounding school leadership, institutional response, education authorities, and public officials. These aspects emerged as important focal points in the escalation of public criticism. The cook's isolated public apology, combined with officials' early departure from the parent meeting, became pivotal turning points because these actions were widely read as signals of misplaced accountability and insincere institutional posture. Once the narrative of the cook as a scapegoat took hold, public concern shifted toward whether accountability was being assigned to an actor with limited control over procurement and oversight rather than to those positioned higher in the responsibility chain. Research on hierarchical governance suggests that, under such conditions, public attention often becomes increasingly focused on procedural and distributive justice (Chan et al., 2023; Li et al., 2021), a pattern that was also evident in the public discourse examined in this case.

Response posture itself becomes a central component of crisis evaluation. When responsibility allocation is perceived as symbolic rather than substantive, negative sentiment is more likely to shift from criticism of the incident itself to critical evaluations of institutional conduct. Viewed through the lens of crisis politicization, the central risk in high-attention crises lies not only in the incident itself, but in whether the public accepts how responsibility is defined and enacted. Discussions around capital conflict, fee disputes, school–enterprise tensions, and operational management suggest that negative sentiment had expanded into broader concerns about structural fairness. The contrast between high educational costs and poor cafeteria management pushed discussion beyond the immediate food safety case and toward broader questions of distributive fairness in educational service provision. Expectation disconfirmation research suggests that public evaluations of institutional performance depend not only on absolute performance, but on the gap between institutional performance and prior expectations (Petrovsky et al., 2017; Van Ryzin, 2004, 2006). When educational costs are perceived to be sharply misaligned with management responsibility and service quality, public discussion appeared more likely to extend beyond the specific incident toward broader concerns about governance fairness. This pattern, observed in the present case, is consistent with expectation disconfirmation research suggesting that such gaps broaden the way governance failures are interpreted by the public.

In the later stages, penalties and accountability outcomes no longer generated peak emotional reactions, but instead produced a more durable undercurrent of low-intensity negativity. Public criticism was directed less at punishment itself than at whether penalties were proportionate to the severity of the incident and whether accountability extended to key decision-making levels. Procedural justice research suggests that when sanctions are perceived as proportionate and accountability mechanisms as fair, the public is more likely to recognize institutional legitimacy (Jackson et al., 2012; Sunshine and Tyler, 2003). Compared with earlier emotional peaks, this later-stage negativity appeared less as an immediate emotional reaction and more as a sustained critical evaluation of accountability outcomes.

Taken together, negative sentiment in school food safety crises does not emerge from a single trigger, but accumulates across multiple governance nodes, including food safety violations, response failures, structural fairness concerns, and accountability deficiencies. This pattern further suggests that, in preventable crises characterized by high levels of attributed responsibility, negative evaluative signals are more likely to intensify during post-incident response, explanation, and accountability processes than from the triggering incident alone. Rather than remaining fixed to the incident itself, negative public sentiment appeared to be continually reorganized through evolving judgments about responsibility, response posture, and accountability arrangements, reflecting the stage-sensitive nature of public evaluations during crises.

### 5.3 Positive sentiment: conditional positive evaluations following regulatory intervention and remediation

Compared with the intense concentration of negative sentiment during the acute crisis stage, the remediation and early recovery period showed some easing of public emotion. Aspects related to market regulation, quality control, and broader social rectification attracted comparatively higher levels of positive sentiment. This finding indicates that relatively more favorable public evaluations were observed in discussions surrounding visible regulatory intervention and corrective actions. From a temporal perspective, positive sentiment tended to emerge after several key governance moments, such as the release of joint investigation findings, dedicated remediation meetings, or additional directives from higher-level authorities. These patterns align with prior crisis communication research on corrective action and are also compatible with SCCT's repair logic in high-responsibility crises. Prior research suggests that timely, visible, and relatively transparent remedial measures rarely erase earlier negative attributions but may contribute to more favorable public evaluations following crisis response (Hirschfeld and Thielsch, 2022; Zhu et al., 2020). The sentiment patterns observed in the present case are broadly consistent with this interpretation.

However, this positivity was clearly conditional rather than stable. Public approval was directed more toward the visible initiation of corrective action than toward the overall crisis response, and it should not be interpreted as broad endorsement of institutional performance. Against the strong negative impressions generated earlier by food safety failures, inappropriate response posture, and accountability disputes, positive sentiment during remediation remained limited in both scope and duration, leaving the overall sentiment distribution predominantly negative throughout the remediation stage. The findings further suggest that isolated corrective actions were accompanied by only modest shifts in sentiment distribution during the observation period. Sustained information disclosure also appears more effective than one-time public statements in reducing uncertainty and improving public evaluations (Porumbescu et al., 2017; Worthy, 2015). The durability of positive sentiment may depend on whether subsequent governance remains visible during remediation. Where disclosure is inadequate, remediation lacks clarity, or institutional reform remains superficial, the limited positive evaluations observed during remediation may not prove durable.

In school food safety crises, such positive evaluations are better understood as conditional signals of institutional repair associated with regulatory intervention and corrective action rather than as comprehensive endorsements of institutional performance. Rather than wholly rejecting governing institutions, the sentiment distribution suggests that public discussion continued to focus on whether meaningful institutional improvement would follow. The observed shift from concentrated negative evaluations toward more balanced sentiment distributions suggests that public sentiment evolves in stages alongside governance developments rather than remaining locked in a single emotional state. More durable positive evaluations were observed primarily where subsequent governance remained visible and continuous. In highly sensitive public service crises, remediation may provide short-term buffering; sustained favorable evaluations, however, appear closely associated with the continuity and verifiability of post-crisis institutional action.

### 5.4 Relatively balanced sentiment distribution: procedural observation and conditional institutional expectations

Six governance aspects—official investigation, official notification, legal baseline, supply chain, responsibility implementation, and regulatory mechanisms—exhibited relatively balanced sentiment distributions (Cohen's *h* < 0.2) during the later stages of the crisis, indicating only small differences between the proportions of positive and negative evaluations. Although these aspects remained slightly negative overall, they differed markedly from the concentrated negativity observed during the acute stage. Rather than simply extending the earlier concentration of negative evaluations, this pattern suggests that public discussion gradually shifted toward procedural evaluation, with immediate reactions to the incident giving way to closer assessment of governance processes.

This pattern should not be interpreted as an absence of public judgment; rather, it reflects a provisional evaluative stance characterized by observation, caution, and waiting. Official investigation illustrates this clearly. Much of the discussion expressed approval because formal investigation signaled that accountability processes and legal procedures were now underway. Yet such approval should not be interpreted as a comprehensive positive evaluation of institutional performance. During periods when investigations appeared to progress slowly or disclosure remained limited, dissatisfaction resurfaced in public discussion. Official notification followed a similar pattern, with public evaluations appearing closely associated with both the substance and timeliness of the information provided. Notifications perceived as delayed or lacking meaningful detail tended to be accompanied by more skeptical public responses, and were sometimes framed in online discussion as indicators of institutional opacity. In the later crisis stage, discussion surrounding institutions became relatively more balanced when procedural actions were visibly implemented, but such evaluations depended on whether those procedures were substantive and credible rather than merely symbolic. From the perspective of public evaluations across crisis stages, public attention appeared to shift from early-stage responsibility attribution toward whether institutional procedures appeared substantive and credible.

Debates over supply chains, regulatory mechanisms, and responsibility implementation further reflected a shift from case-specific reactions toward broader systemic reflection. Rather than rejecting institutions wholesale, public concern centered more on the rigor of enforcement, the completeness of oversight systems, and whether similar failures could be prevented in the future. Prior research suggests that public evaluations are shaped not only by immediate crisis performance but also by judgments about institutional self-correction and procedural reliability (Van der Meer and Zmerli, 2017).

Seen in this light, a relatively balanced sentiment distribution reflects an ongoing process of public evaluation rather than a stable positive assessment. At this stage, positive and negative evaluations coexist, suggesting that public attention has shifted from immediate reactions to continued observation of institutional responses and governance processes. The persistence of relatively balanced distributions suggests that public evaluation at this stage remained contingent on the quality and continuity of subsequent governance, rather than having resolved into stable positive assessment. In school food safety crises, later-stage public sentiment need not remain confined to simple support or opposition; it may instead take the form of a transitional condition centered on procedural scrutiny of institutional action.

### 5.5 Theoretical and methodological contributions of the topic–aspect–sentiment framework

The methodological value of this study lies in embedding sentiment analysis within structured governance issues, thereby revealing the differentiated logic underlying public evaluations. Through issue-level refinement, the framework identifies differences in evaluative patterns across governance nodes within a single crisis event—differences that aggregate sentiment polarity statistics would typically reduce to a single average, thereby obscuring the distinct accountability logics and institutional evaluation standards embedded in each issue. In highly sensitive public events such as school food safety crises, where multiple governance dimensions are closely intertwined, issue-level differentiation is not simply an analytical refinement but is essential for identifying how public evaluations differ across governance issues. In this sense, the Topic–Aspect–Sentiment framework offers a transferable analytical template for studying similar high-sensitivity public service crises. It is particularly suited to contexts involving multiple governance actors, stage-based sentiment dynamics, and highly issue-specific public judgments. Its boundaries and conditions of transferability, however, require further validation through cross-case research. The findings should also be interpreted in light of Weibo's specific communication environment. On this platform, content visibility and reach are shaped not only by user interaction but also by recommendation algorithms, trending topic mechanisms, and content moderation rules. The issue structures and sentiment patterns identified in this study therefore reflect not only how the public evaluated the crisis itself, but also, to some extent, how relevant information was surfaced and circulated on the platform. Public sentiment on social media should accordingly be understood as the product of both public evaluation and platform-level conditions, rather than as a direct reflection of the full range of public opinion.

## 6 Conclusions and practical implications

### 6.1 Research conclusions

Using a school food safety incident in Yunnan Province as a case study, this research draws on Weibo data and combines manual sentiment annotation, LDA topic modeling, and aspect-level analysis to develop and apply a Topic–Aspect–Sentiment framework for examining issue-specific sentiment distributions and their stage-based evolution in school food safety crises. The findings show that public evaluations in school food safety crises exhibit clear issue-specific patterns and stage-dependent variations across governance issues. Sentiment was not distributed evenly, but instead clustered around governance issues such as food safety violations, response posture, responsibility attribution, and perceptions of accountability fairness. As the crisis evolved, public evaluation shifted from immediate concern over the incident itself toward sustained scrutiny of governance processes. The findings therefore primarily reflect how public evaluations varied across governance issues rather than how institutional trust changed over time. In preventable crises of this kind, variations in public evaluations were observed not only in relation to the triggering event itself but also alongside subsequent institutional responses and responsibility management.

First, the study moves beyond aggregate sentiment trends by locating public evaluation within specific governance issues, revealing how sentiment is distributed differently across topics and crisis stages and offering finer-grained empirical insight into sentiment formation within a single crisis. Second, Methodologically, the Topic–Aspect–Sentiment framework also offers a way to translate social media texts into observable signals of crisis stages. This study treats online sentiment as an evaluative signal through which the public expresses judgments about governance issues and institutional performance rather than as a direct measure of trust or institutional trustworthiness. The findings should therefore be interpreted within this scope. Third, the study empirically identifies the governance domains most vulnerable to concentrated negative evaluation in school food safety crises, offering practical guidance for communication strategy design in comparable cases.

As this study draws on a single case and a single platform, its conclusions carry inherent contextual limitations. Weibo has a distinct user base and content ecosystem and occupies a unique position within China's media landscape. Differences across platforms in user demographics, content moderation norms, and recommendation algorithms logic directly shape what users post and what they are likely to encounter. Future research could compare the evolution of public evaluations across platforms during similar crisis events to examine whether the sentiment patterns identified here reflect broadly shared public attitudes or are partly shaped by Weibo's distinctive crisis communication environment. Further research could also examine how recommendation algorithms, trending topic mechanisms, and content governance rules shape issue visibility and sentiment expression across platforms. In addition, while manual coding preserves contextual sensitivity, it is constrained by limitations in coding scale and by the inherent subjectivity of judgment. Future research could explore integrated approaches that combine manual coding with more refined automated sentiment classification, in order to increase analytical scale while retaining interpretive validity. Although this study uses publicly available text data and applies anonymization, research involving social media content still requires attention to ethical boundaries, which has partially limited the presentation of certain contextual details. The findings should be understood as reflecting public evaluations as expressed through social media discourse. Although issue-level sentiment distributions clarify how the public evaluates institutional behavior and governance processes, they should not be interpreted as direct evidence of changes in institutional trust or related trust perceptions. Future studies will need to incorporate direct measures of trust to examine these relationships more explicitly.

### 6.2 Implications for crisis communication and governance

Building on these findings, public evaluations in school food safety crises are shaped not only by the incident itself, but also by information transparency, response conduct, accountability communication, and shifting governance priorities across crisis stages. Accordingly, this study offers practical implications in four key areas.

First, crisis governance requires transparency throughout the entire information disclosure process, rather than limiting communication to initial notification. Although sentiment surrounding official notification, official investigations, and remediation became more moderated in later stages, negative evaluations resurfaced whenever updates were delayed, vague, or unsupported by meaningful follow-through. Public concern therefore centers not simply on whether institutions respond, but on whether crisis information remains continuously visible and whether governance progress can be transparently tracked. In high-accountability events such as school food safety crises, information disclosure should span the entire crisis process, ensuring that investigation progress and remediation implementation remain traceable. Sustained, clear, and verifiable disclosure is more likely to reduce uncertainty and convey that governance efforts are continuing in substantive ways.

Second, response posture should be treated as a central component of crisis governance rather than as a secondary tool for post-crisis repair. During the acute crisis stage, response failures were associated with more concentrated negative sentiment than the triggering incident itself. This pattern was particularly evident when accountability attribution appeared misaligned with the actual chain of responsibility, allowing criticism to extend beyond the specific incident toward broader evaluations of institutional conduct and governance performance. Consistent with SCCT, high-responsibility crises are particularly vulnerable to escalating negativity when institutions adopt defensive or evasive responses, whereas visible acceptance of responsibility is more likely to contain attributional escalation. For schools and relevant governance actors, this means responsibility communication must be both timely and structurally credible. At key governance moments, institutions should clarify responsibility boundaries, explain decision rationales, and ensure that communication is led by actors with genuine decision-making authority. Avoiding symbolic blame placed solely on frontline personnel with limited structural control is particularly important, as misdirected accountability can intensify rather than reduce public controversy.

Third, public evaluations of accountability and remediation depend heavily on whether the rationale behind crisis management is transparent and understandable to the public. In later crisis stages, public attention often shifts from whether punishment occurs to whether it is fair, proportionate, and capable of producing meaningful institutional reform. Public dissatisfaction was therefore not reducible to perceptions of lenient punishment alone. More deeply, uncertainty centered on whether sanctions were fairly assigned, whether accountability levels were appropriate, what standards guided judgment, and whether remediation would carry meaningful institutional force. Under such conditions, formulaic statements that a matter has been handled according to regulations may do little to reduce skepticism and may instead reinforce perceptions of procedural evasion. When announcing outcomes, schools and regulatory authorities should therefore move beyond declarative conclusions by clearly explaining the scope of responsibility, the basis for sanctions, and the concrete institutional reforms intended to follow. Negative evaluations are more likely to moderate when crisis communication demonstrates a genuine institutional commitment to addressing the crisis and when case-specific responses are visibly linked to broader structural improvements.

Fourth, crisis communication strategies should adapt across different stages rather than relying on uniform approaches throughout the process. The findings show that public evaluative standards differ across stages: in the initial stage, attention centers on factual accuracy; in the acute stage, on responsibility attribution and response attitude; in the remediation stage, on whether corrective actions are genuinely advancing; and in the later stage, on whether institutional repair is sustained and verifiable. This means that effective crisis governance requires not only sustained responses, but continuous adjustment of response priorities in line with shifts in public attention. Institutions that apply the same communication logic throughout the crisis are unlikely to meet the public's evolving governance expectations. For school administrators and local governance actors, a more effective strategy is to tailor crisis communication to three stage-specific priorities: fact-based communication, action-oriented response, and long-term governance improvement. This approach treats crisis communication as a dynamic process spanning the full crisis life cycle rather than confining it to immediate response during the outbreak stage.

## Data Availability

The data were obtained from the Sina Weibo platform and are subject to platform privacy policies and user agreements. Processed and anonymized datasets supporting the conclusions of this study are available from the corresponding author upon reasonable request.
